# Metal Oxide Gas Sensors: Sensitivity and Influencing Factors

**DOI:** 10.3390/s100302088

**Published:** 2010-03-15

**Authors:** Chengxiang Wang, Longwei Yin, Luyuan Zhang, Dong Xiang, Rui Gao

**Affiliations:** Key Laboratory for Liquid-Solid Structural Evolution and Processing of Materials, Ministry of Education, Shandong University, Jinan, 250061, China; E-Mails: xiaowang412@163.com (C.W.); 107250458@qq.com (L.Z.); gaorui1985@gmail.com (R.G.)

**Keywords:** metal oxide, gas sensors, sensitivity, surface reaction

## Abstract

Conductometric semiconducting metal oxide gas sensors have been widely used and investigated in the detection of gases. Investigations have indicated that the gas sensing process is strongly related to surface reactions, so one of the important parameters of gas sensors, the sensitivity of the metal oxide based materials, will change with the factors influencing the surface reactions, such as chemical components, surface-modification and microstructures of sensing layers, temperature and humidity. In this brief review, attention will be focused on changes of sensitivity of conductometric semiconducting metal oxide gas sensors due to the five factors mentioned above.

## 1. Introduction

The conductometric semiconducting metal oxide gas sensors currently constitute one of the most investigated groups of gas sensors. They have attracted much attention in the field of gas sensing under atmospheric conditions due to their low cost and flexibility in production; simplicity of their use; large number of detectable gases/possible application fields. In addition to the conductivity change of gas-sensing material, the detection of this reaction can be performed by measuring the change of capacitance, work function, mass, optical characteristics or reaction energy released by the gas/solid interaction [1]. As a simple review of metal oxide gas sensors, the main attention in this paper will be focused on the conductometric semiconducting metal oxide gas sensors (especially surface conductive metal oxide).

Numerous researchers have shown that the reversible interaction of the gas with the surface of the material is a characteristic of conductometric semiconducting metal oxide gas sensors [1]. This reaction can be influenced by many factors, including internal and external causes, such as natural properties of base materials, surface areas and microstructure of sensing layers, surface additives, temperature and humidity, *etc.* Many papers about metal oxide gas sensors have been published in recent years [1–20]. As one of the important parameters of gas sensors, sensitivity has been attracting more and more attention and much effort has been made to enhance the sensitivity of gas sensors. There is not a uniform definition for gas sensor sensitivity now. Usually, sensitivity (S) can be defined as Ra/Rg for reducing gases or Rg/Ra for oxidizing gases, where Ra stands for the resistance of gas sensors in the reference gas (usually the air) and Rg stands for the resistance in the reference gas containing target gases. Both Ra and Rg have a significant relationship with the surface reaction(s) taking place. Although there are many reviews in this field, to the best of our knowledge there were no special reviews about the factors influencing sensitivity. In this paper, we have thus focused on a brief survey of the effect of the surface reaction factors on the sensitivity.

## 2. Metal Oxides for Gas Sensors

Many metal oxides are suitable for detecting combustible, reducing, or oxidizing gases by conductive measurements. The following oxides show a gas response in their conductivity: Cr_2_O_3_, Mn_2_O_3_, Co_3_O_4_, NiO, CuO, SrO, In_2_O_3_, WO_3_, TiO_2_, V_2_O_3_, Fe_2_O_3_, GeO_2_, Nb_2_O_5_, MoO_3_, Ta_2_O_5_, La_2_O_3_, CeO_2_, Nd_2_O_3_ [21]. Metal oxides selected for gas sensors can be determined from their electronic structure. The range of electronic structures of oxides is so wide that metal oxides were divided into two the following categories [1]:

1. Transition-metal oxides (Fe_2_O_3_, NiO, Cr_2_O_3_, *etc.*)
2. Non-transition-metal oxides, which include (a) pre-transition-metal oxides (Al_2_O_3_, *etc.*) and (b) post-transition-metal oxides (ZnO, SnO_2_, *etc.*).

Pre-transition-metal oxides (MgO, *etc.*) are expected to be quite inert, because they have large band gaps. Neither electrons nor holes can easily be formed. They are seldom selected as gas sensor materials due to their difficulties in electrical conductivity measurements. Transition-metal oxides behave differently because the energy difference between a cation d^n^ configuration and either a d^n+1^ or d^n−1^ configurations is often rather small [22]. They can change forms in several different kinds of oxides. So, they are more sensitive than pre-transition-metal oxides to environment. However, structure instability and non-optimality of other parameters important for conductometric gas sensors limit their field of application. Only transition-metal oxides with d^0^ and d^10^ electronic configurations find their real gas sensor application. The d^0^ configuration is found in binary transition-metal oxides such as TiO_2_, V_2_O_5_, WO_3_. d^10^ configuration is found in post-transition-metal oxides, such as ZnO, SnO_2_.

## 3. Sensing Mechanism

**Figure 1.**
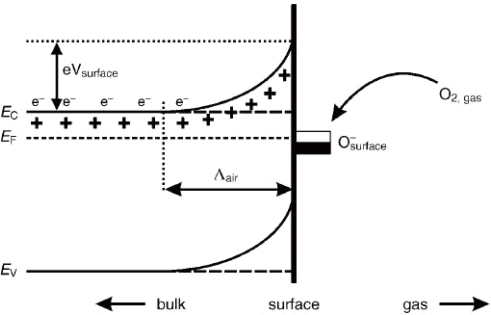
Schematic diagram of band bending after chemisorptions of charged species (here the ionosorption of oxygen) E_C_, E_V_, and E_F_ denote the energy of the conduction band, valence band, and the Fermi level, respectively, while Λair denotes the thickness of the space-charge layer, and eV_surface_ denotes the potential barrier. The conducting electrons are represented by e^−^ and + represents the donor sites (adapted from [23]).

**Figure 2.**
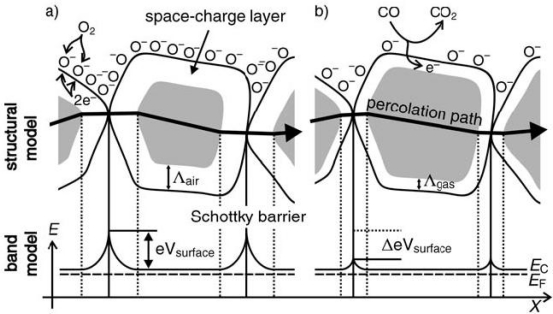
Structural and band models of conductive mechanism upon exposure to reference gas. (a) with or (b) without CO (adapted from [23,24]).

Considering the influence factors on gas sensing properties of metal oxides, it is necessary to reveal the sensing mechanism of metal oxide gas sensor. The exact fundamental mechanisms that cause a gas response are still controversial, but essentially trapping of electrons at adsorbed molecules and band bending induced by these charged molecules are responsible for a change in conductivity. The negative charge trapped in these oxygen species causes an upward band bending and thus a reduced conductivity compared to the flat band situation. As shown in Figure 1 [23], when O_2_ molecules are adsorbed on the surface of metal oxides, they would extract electrons from the conduction band Ec and trap the electrons at the surface in the form of ions. This will lead a band bending and an electron-depleted region. The electron-depleted region is so called space-charge layer, of which thickness is the length of band bending region. Reaction of these oxygen species with reducing gases or a competitive adsorption and replacement of the adsorbed oxygen by other molecules decreases and can reverse the band bending, resulting in an increased conductivity. O^−^ is believed to be dominant at the operating temperature of 300–450 °C [5] which is the work temperature for most metal oxide gas sensors. Figure 2 schematically shows the structural and band model of conductive mechanism upon exposure to reference gas with or without CO When gas sensors exposure to the reference gas with CO, CO is oxidized by O^−^ and released electrons to the bulk materials. Together with the decrease of the number of surface O^−^, the thickness of space-charge layer decreases. Then the Schottky barrier between two grains is lowered and it would be easy for electrons to conduct in sensing layers through different grains. However, the mechanism in Figure 1 is only suitable for n-type semiconducting metal oxides of which depletion regions are smaller than grain size.

## 4. Factors Influencing the Sensitivity

### 4.1. Chemical Composition

Semiconducting metal oxides have been investigated extensively at elevated temperatures for the detection of simple gases. [25] There are many parameters of materials for gas sensor applications, for example, adsorption ability, catalytic activity, sensitivity, thermodynamic stability, *etc.* Many different metal oxide materials appear favorable in some of these properties, but very few of them are suitable to all requirements. For this situation, more recent works focus on composite materials, such as SnO_2_-ZnO [26,27] Fe_2_O_3_-ZnO [28], ZnO-CuO [29] *etc.* In addition to binary oxides, there are numerous ternary, quaternary and complex metal oxides, which are of interest of mentioned applications [30,31]. The combination of metal oxides and other components, for example, organic and carbon nanotubes, were also investigated much. Herein, we mainly take composite metal oxides as examples to introduce the influence of chemical composition.

**Figure 3.**
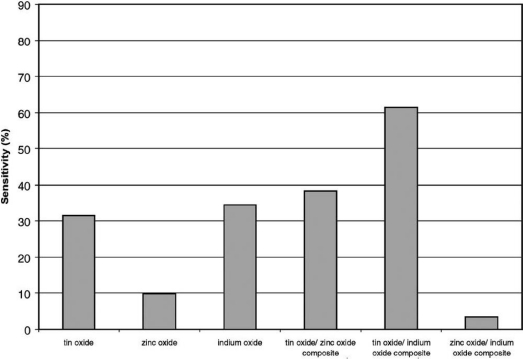
The response of single oxide and composite sensors to 5 ppm ethanol vapour at 100% RH (adapted from [32]).

The composite ZnO-SnO_2_ sensors exhibited significantly higher sensitivity than sensors constructed solely from tin dioxide or zinc oxide when tested under identical experimental conditions [28]. Sensors based on the two components mixed together are more sensitive than the individual components alone suggesting a synergistic effect between the two components. Details about the synergistic effect is still unknown, but de Lacy Costello and co-workers [28] have suggested a possible mechanism. Taking SnO_2_-ZnO binary oxides responding to butanol as an example, they hypothesize that butanol is more effectively dehydrogenated to butanal by tin dioxide, but that tin dioxide is relatively ineffective in the catalytic breakdown of butanal. On the other hand, zinc oxide catalyses the breakdown of butanal extremely effectively. A combination of the two materials would effectively dehydrogenate butanol and then subsequently catalyse the breakdown of butanal. The catalysis results obtained when using the composite support this idea. This explanation suggests that not all composite gas sensors will have better performances than those of individual components alone. Only when the catalytic action of the components complements each other, the performance of gas sensors will be enhanced. As shown in Figure 3 [32], composites of tin dioxide/zinc oxide and tin dioxide/indium oxide display enhanced sensitivity when compared with the single oxide sensors. However, composite sensors comprising mixtures of zinc oxide and indium oxide show a reduction in sensitivity when compared directly with the equivalent single oxide sensors.

**Figure 4.**
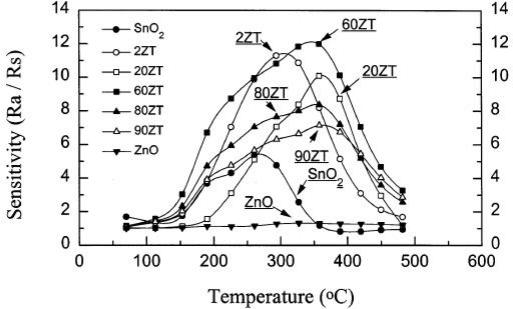
Temperature dependence of CO sensitivity (200 ppm) of SnO_2_, ZnO and ZnO-SnO_2_ composites. 20 ZT means 20 mol% ZnO–80 mol% SnO_2_ sample and others are the similar (adapted from [26]).

In addition to the synergistic effect, heterojunction interface between two or more components also contributes to the enhancement of the composite gas sensor performance [33–38]. The principle of formation of heterojunction barriers in air ambient and their disruption on exposure to target gas is employed. So, the resistance and proportion of p-n heterojunctions in the composite gas sensor becomes a control factor to the gas sensor performance. Furthermore, it has shown that changing the proportions of each material in the composite yields a wide range of sensor materials with very different sensing characteristics. Figure 4 shows the temperature dependence of CO sensitivity (200 ppm) of SnO_2_, ZnO and ZnO-SnO_2_ composites. We can see that different addition of ZnO to SnO_2_ leads to different responses to ethanol.

### 4.2. Surface Modification by Noble Metal Particles

In many gas sensors, the conductivity response is determined by the efficiency of catalytic reactions with detected gas participation, taking place at the surface of gas-sensing material. Therefore, control of catalytic activity of gas sensor material is one of the most commonly used means to enhance the performances of gas sensors. However, in practice, the widely used gas sensing metal oxide materials such as TiO_2_, ZnO, SnO_2_, Cu_2_O, Ga_2_O_3_, Fe_2_O_3_, are the least active with catalytic point of view [1]. The pure SnO_2_ thin film without any catalyst exhibits a very poor sensitivity (∼3) confirming this statement [39].

**Figure 5.**
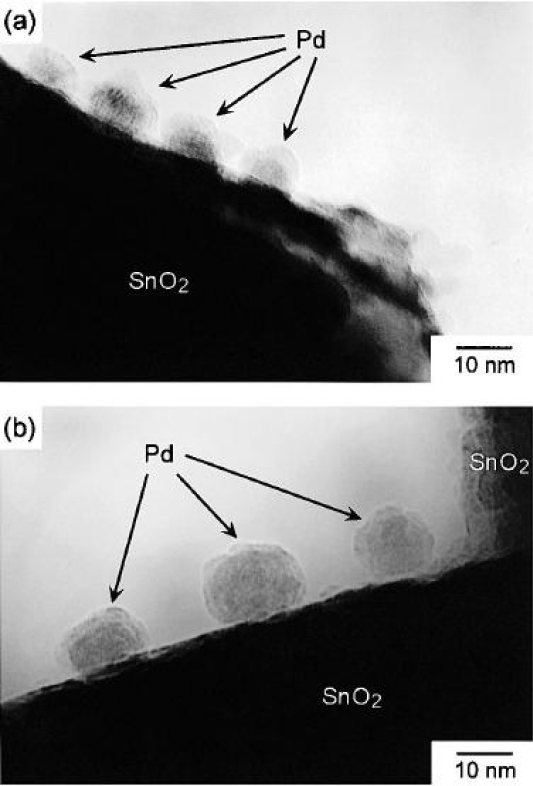
Two representative TEM photographs of Pd particles loaded on the surface of SnO2 (adapted from [40]).

**Figure 6.**
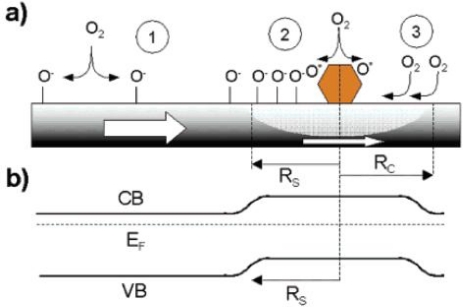
(a) Schematic depiction of the major process taking place at a SnO_2_ nanowire/nanobelt surface when exposured to O_2_. (b) Band diagram of the pristine SnO_2_ nanostructure and in the vicinity (and beneath) a Pd nanoparticle. The radius of the depletion region is determined by the radius of the spillover zone (adapted from [46]).

Noble metals are high-effective oxidation catalysts and this ability can be used to enhance the reactions on gas sensor surfaces. A wide diversity of methods, including impregnation, sol-gel, sputtering and thermal evaporation, has been used for introducing noble metal additives into oxide semiconductors. Different doping states can be obtained by different methods. Mixture of noble metal particles and metal oxides may be obtained by sol-gel method, while metal oxides modified by noble metal particles on the surface are possibly obtained by sputtering or thermal evaporation. This section will focus on the latter. Two representative TEM images of Pb-modified SnO_2_ are shown in Figure 5 [40]. There have been many reports for enhancement of sensitivity modified by noble metals, such as Pt, Au, Pd, Ag, *etc.* [41–45]. In [46], the authors introduced the mechanism of this method in details. It can be described generally as follows:

Commonly, two concepts are invoked to explain the improvement of the nanowire’s sensing performance upon Pd deposition. One is the “electronic mechanism” proposal and the other is a “chemical” process. The “electronic mechanism” considered that depletion zones formed around the modified particles (Figure 6b) and the modulation of the nano-Schottky barriers (and hence the width of the conduction channel) which is due to changes in the oxidation state of the Pd accompanying oxygen adsorption and desorption is responsible for the sensing enhancement. However, the “electronic mechanism” has some difficulties in explaining the kinetics and temperature dependence brought about by Pd functionalization, while the “chemical” process not. The latter mechanism bases on the highly effective dissociation catalytic ability of Pd. The ionosorption of oxygen at defect sites of the pristine surface is shown in Figure 6a process-1. Pd is a far better oxygen dissociation catalyst than tin oxide and catalytically activate the dissociation of molecular oxygen in process one. Then atomic products diffuse to the metal oxide support as shown in Figure 6a process-2. Furthermore, it is not necessary for molecular oxygen to dissociate on Pd surface only in process two. It is believed that oxygen molecules can reside briefly on an oxide support and diffuse to a catalyst particle before it has had an opportunity to desorb [47]. This is the so-called “back-spillover effect” (procees-3 in Figure 6a).There is an effective “capture radius” (Rc in Figure 6b) around the Pd particles. An effective oxygen delivery system forms if the whole surface of metal oxide covered by this oxygen “collect zones” [48]. The net results of process-2 and -3 significantly enhance the probability of oxygen ionosorption on metal oxides and hence the sensitivity. The interaction of reducing gas such as H_2_, with pristine and Pd-functionalized SnO_2_ was thoroughly discussed in [49] and the principle transduction mechanism is similar to that of O_2_ mentioned above. However, the dispersion of catalysts is also an important factor to develop the potential of catalysts. So, the structure of catalyst supporting materials would be considered in the next step.

### 4.3. Microstructure

The operating characteristics of solid state gas sensors are determined by both receptor and transducer functions. The last function is very important, because it determines the efficiency of chemical interactions’ conversion into electrical signal. Therefore, it is very important to synthesize metal oxides with optimal morphology and crystallographic structure.

**Figure 7.**
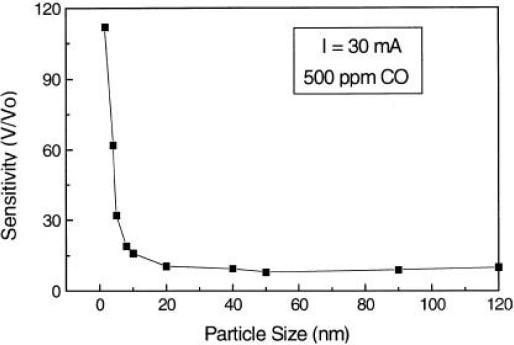
Effect of particle size on gas sensitivity for CO (adapted from [50]).

**Figure 8.**
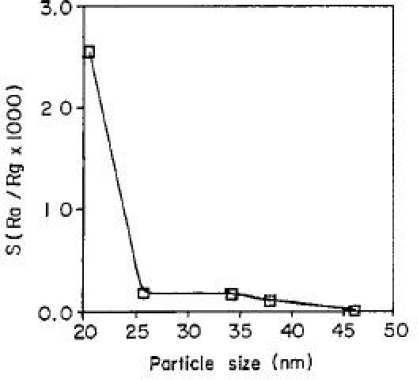
Variation in sensitivity with average particle size (adapted from [51]).

A sensor’s sensitivity can be significantly increased by using materials with very small grains sixes, and this simulated result agrees well with the experimental observation. Lu *et al.* [50] have indicated that the SnO_2_-based sensor response to 500 ppm CO increases drastically if the particle diameter becomes smaller than 10 nm as shown in Figure 7. Studies of SnO_2_ nanoparticle sensor layers exposure to H_2_ in Reference [51] indicated that gas response using 20 nm particles was about 10 times more sensitive than that of using 25∼40 nm particles as shown in Figure 8. For small grains and narrow necks, when the grain size is less than twice the thickness of surface charge layers, the grain is fully involved in the space-charge layer [3]. Then a surface influence on free charge carrier’s mobility should be taken into consideration. This happens because the number of collisions experienced by the free charge carriers in the bulk of the grain becomes comparable with the number of surface collisions. The latter may be influenced by adsorbed species acting as additional scattering centers [52]. This case is most desirable because the sensitivity of metal oxide gas sensors can be enhanced significantly by this method. More details can be seen in reference [52–56]. It is necessary to note that smaller crystal size of nanocrystals does not necessarily mean the enhancement in gas response. Studies in Reference [57] indicated that the sensor sample based on SnO_2_ nanocrystals (∼50 nm) produced by gel combustion method had higher response and shorter response time than the one prepared from hydrothermal-synthesized SnO_2_ nanocrystals (12–13 nm). It can be attributed to the more porous nanocrystalline microstructure of SnO_2_ nanocrystals produced by gel combustion method than that offered by hydrothermal-synthesized SnO_2_ nanocrystals which are smaller in size but tend to agglomerate into large entities. This is a limit in the process of enhancing performances of metal oxide gas sensors by decreasing the grain size. Finely dispersed small crystallites will have a deleterious effect on the temporal stability of the sensor. An excessive decrease of grain size leads to a loss of structural stability [58], and, as a consequence, to change both surface and catalytic properties of the material [59].

**Figure 9.**
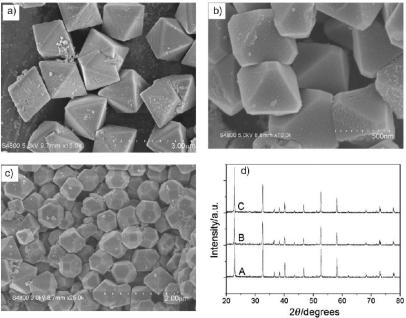
Typical SEM images of the as-prepared ZnSnO_3_ products: (a) CTAB = 0.15 M; (b) CTAB = 0.4 M; (c) CTAB = 0.75 M. (d) The corresponding XRD patterns of the as-prepared ZnSnO_3_ polyhedra, A: octahedra; B: truncated octahedra; C: 14-faceted polyhedra (adapted from [60]).

**Figure 10.**
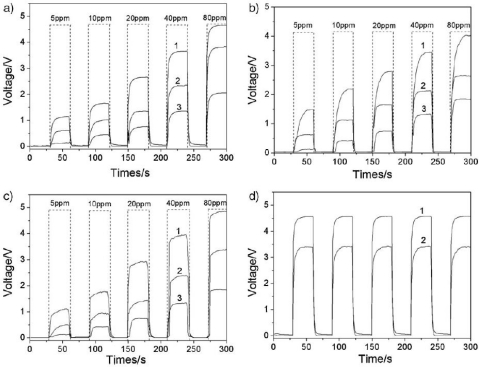
Typical response curves of 14-faceted polyhedral (line 1) and octahedral (line 2) ZnSnO_3_ and ZnSnO_3_ powder (line 3) gas sensors to (a) H_2_S, (b) HCHO, (c) C_2_H_5_OH with increasing concentrations. (d) The response curves of 14-faceted polyhedra (line1) and octahedra (line 2) to H_2_S (70 ppm) at room temperature (adapted from [60]).

The controlled synthesis of nano- or microsized particles with different shape and morphology has attracted considerable interest, because the properties of nano- and microcrystals depend not only on their composition, but also on their structure, phase, shape, size, and size distribution. The reactivity and selectivity of a nanocatalyst can be tailored by controlling the shape, as it will determine the crystallographic facets exposed on the surface of a nanocrystal and therefore the number of atoms located at the edges or corners. In [60], uniform octahedral, truncated octahedral, and 14-faceted polyhedral ZnSnO_3_ microcrystals was prepared through addition of different amount of sodium dodecylbenzene sulfonate (SDBS) (Figure 9) and gas responses exposure to H_2_S, HCHO and C_2_H_5_OH were tested (Figure 10). The results indicated that the 14-faceted polyhedral ZnSnO_3_ has a higher sensitivity than the octahedral, due to a larger active surface area of {100} facets, which can provide more active space for the interaction between ZnSnO_3_ and target gases.

**Figure 11.**
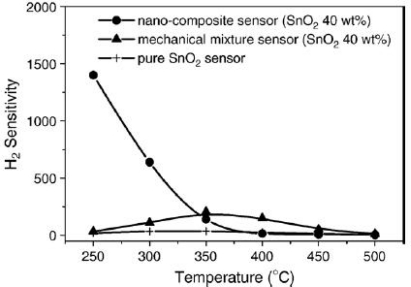
Correlation between sensing temperature and sensitivity to 1,000 ppm H_2_ of different types of SnO_2_ gas sensors (adapted from [65]).

Besides the activation of the crystallographic facets exposure to target gases, high surface to volume ratio is also crucial as mentioned above. However, high surface to volume ratio can be obtained not only by reducing grain size, but also by highly-ordered pore structure such as mesoporous structure or nanotube arrays. The very high surface to volume ratio of mesoporous materials has attracted much attention in the application of gas sensors [61,62]. However, the most of such mesoporous structured metal oxides are not stable after the removal of surfactant and pore structure would collapse at high temperature [63,64]. High surface areas make them excellent supporting materials, which can obtain well-dispersed gas sensor materials or catalysts. Figure 11 shows the sensitivities of SnO_2_ (40 wt%) /SBA-15 composite sensor and pure SnO_2_ sensor to 1,000 ppm of H_2_ [65]. The SnO_2_ (40 wt%)/ SBA-15 sensor displays a much enhancement of sensitivity to H_2_, especially at 250 °C where the sensitivity of the sensor to 1,000 ppm of H_2_ reaches as high as 1,400, almost 40 times higher than that of the pure SnO_2_ sensor. Furthermore, the optimum sensing temperature of SnO_2_ (40 wt %) /SBA-15 sensor where the sensitivity is maximum is around 250 °C, which is lower than that of the pure SnO_2_ sensor (350 °C). The authors considered that SBA-15 is a good adsorbent for gases [66], which can make the nano-composite sensor adsorb more oxygen or hydrogen and promote the reaction between hydrogen and the surface adsorbed oxygen. Moreover, the adsorption of gases at lower temperature may be enhanced by the presence of mesoporous SBA-15, the optimum gas sensing temperature would shift to a lower value. Studies on methane sensing properties of a tin oxide-based sensor with alumina supported Pd catalyst indicated that excellent promoting effect of the supported Pd catalyst is considered to originate from the high dispersion of Pd (or PdO) particles on supporting materials [67].

Recent efforts have been focused on the development of nanostructured sensors, taking into account structural peculiarities of gas-sensing materials. One-dimension metal oxides might be perspective enough for fundamental studies as well as for applications due to their structural peculiarities. When the grain size becomes comparable to twice the Debye length, a space-charge region can develop in the whole crystallite as demonstrated in [68] and it allows achievement of maximum sensor response in this case. One-dimension materials can easily be made thin enough comparable to Debye length and most importantly, they should be more thermo-dynamically stable in comparison with nanograins, promoting stable operation of gas sensors at higher temperature over a long time period as demonstrated in reference [69]. One-dimensional metal oxide nanomaterials, such as nanobelts, nanorods, nanowires and so on, show a number of positive features, as facile fabrication, open surface, high gas sensitivity and long-term stability, which make them prospective material platform for the next generation of durable conductometric gas sensors.

### 4.4. Humidity and Temperature

**Figure 12.**
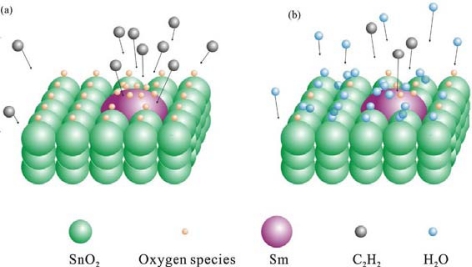
Gas sensing mechanism of Sm_2_O_3_-doped SnO_2_ in the atmosphere of (a) C_2_H_2_ and (b) C_2_H_2_ and humidity (adapted from [74]).

Environmental humidity is an important factor influencing the performance of metal oxide gas sensors, as many humidity gas sensors based on metal oxides have been developed. However, mechanism of sensing water vapor and other pollution gas such as CO, NO_2_, H_2_S, is different. For metal oxide humidity gas sensors, ionic-type humidity sensors are the most common patterns. The conduction mechanism depends on H^+^ or H_3_O^+^, from dissociation of adsorption water, which hops between adjacent hydroxyl groups. Details about the adsorption of water on metal oxide surfaces and mechanism of sensing water vapor can be seen in [70,71]. Water adsorbing on the metal oxide surface will not donate electrons to sensing layers. Moreover, as explained in [72] and [73], it will lower the sensitivity of metal oxide sensors for some reasons as follows. The reaction between the surface oxygen and the water molecules conduces to a decrease in baseline resistance of the gas sensor, and results in a decrease of the sensitivity [73]. Secondly, the adsorption of water molecules leads to less chemisorption of oxygen species on the SnO_2_ surface due to the decrease of the surface area that is responsible for the sensor response. On the other hand, water molecules also act as a barrier against C_2_H_2_ adsorption. The superficial migration of the C_2_H_2_ on the SnO_2_ surface becomes difficult, thus the sensitivity decreases and the response and recovery times increase. Figure 12 shows the schematic of the effect of humidity on the C_2_H_2_ sensing properties.

**Figure 13.**
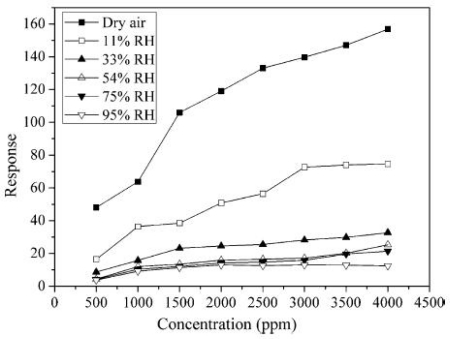
Response of the Sm_2_O_3_-doped SnO_2_ sensor to different concentrations of C_2_H_2_ at different RH (adapted from [72]).

Water adsorption will significantly lower the sensitivity of metal oxide gas sensors, as shown in Figure 13. Furthermore, prolonged exposure to humid environments leads to the gradual formation of stable chemisorbed OH^−^ on the surface [70], causing a progressive deterioration of the sensitivity of gas sensors. However, surface hydroxyls start to desorb at about 400 °C [74] and the hydroxyl ions can be removed by heating to temperatures higher than 400 °C. As shown in [75], after several humidity pulses the sensor resistance does not recover to its initial level, however, subsequent heating up the sensor to a temperature of 450 °C for several minutes leads to a full recovery of the signal. In the conclusions of [45], water strongly inhibits methane combustion even at low partial pressures. However, the effect depends on the reaction temperature, becoming small at temperatures above about 450 °C.

**Figure 14.**
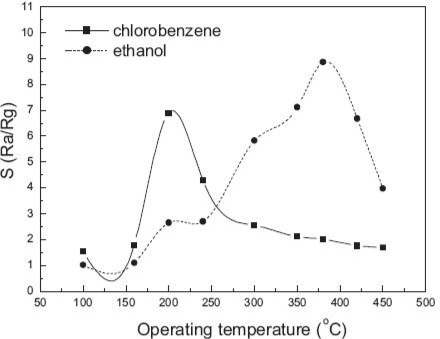
Gas response *versus* operating temperature of porous ZnO nanoplate sensor to 100 ppm chlorobenzene and ethanol (adapted from [76]).

Temperature is also an important factor for the metal oxide gas sensors. Typical curves of gas response *vs*. temperature were shown in Figure 14. Gas sensors with different compositions have similar shapes. The responses increase and reach their maximums at a certain temperature, and then decreased rapidly with increasing the temperature. This tendency is commonly observed in many reports [46,76–80]. In reference [46], the authors considered that the shape resulted from the competition between slow kinetics at low temperatures and enhanced desorption at high temperatures.

## 5. Conclusions

In summary, the gas sensing process is strongly related to the surface reactions. Different metal oxide based materials have different reaction activation to the target gases. Composite metal oxides usually show better gas response than the single component if the catalytic actions of the components complement each other. Noble metal additives with high-effective oxidation catalytic activity can be used to enhance the sensitivity of pure metal oxides due to the “spillover effect”. Moreover, good catalyst supporting materials are also a key point to determine how much potential of catalysts can be developed. So, the structure of metal oxide layers is very important. High surface areas are necessary to obtain highly-dispersed catalyst particles. Furthermore, high surface areas can provide large reaction contact area between gas sensing materials and target gases. Porous structure with high surface areas seems to be the standard structure of metal oxide gas sensor layers. It is assembled by lots of small grains with voids and pores among them. It is also showed that small grain size is useful to enhance the sensitivity. At high temperatures, small grains tend to agglomerate into large entities, decreasing both surface areas and catalytic properties of the material. It is important to keep balance between decreasing grain sizes and stability. Another important structure factor is crystallographic facets. One-dimension materials are prospective material platform for the next generation of durable conductometric gas sensors due to open surface, high gas sensitivity and long-term stability, *etc.* Besides the internal causes of metal oxides mention above, the external causes, such as temperature and humidity, also play an important role in the testing of sensitivity. Humidity will decrease the sensitivity and be harmful to repeatability. Fortunately, it can be eliminated by heating to high temperatures (usually >400 °C).
